# Hyperbaric oxygen therapy affects insulin sensitivity/resistance by increasing adiponectin, resistin, and plasminogen activator inhibitor-I in rats

**DOI:** 10.3906/sag-2011-76

**Published:** 2021-06-28

**Authors:** Cemil KAHRAMAN, Hüseyin YAMAN

**Affiliations:** 1 Department of Biochemistry, Faculty of Health Sciences, Düzce University, Düzce Turkey; 2 Department of Biochemistry, Faculty of Medicine, Karadeniz Technical University, Trabzon Turkey

**Keywords:** Adiponectin, insulin, insulin sensitivity, resistin, plasminogen activator inhibitor-I

## Abstract

**Background/aim:**

Hyperbaric oxygen therapy (HBOT) causes insulin sensitivity, but the reason for this is not known yet. The aim of the present study was to investigate the effect of HBOT on insulin sensitivity via resistin, plasminogen activator inhibitor-I (PAI *-* I), and adiponectin.

**Materials and methods:**

The study was designed using HBOT and control groups, with eight rats in each group. After 20 days of HBOT under 2.5 atmospheres for 90 min, the fasting insulin (FI), resistin, PAI-I, homeostatic model assessment of insulin resistance scores (HOMA–IR), quantitative insulin sensitivity check index (QUICKI), fasting plasma glucose (FPG), triglyceride, and high-density lipoprotein cholesterol (HDL-C) in the plasma were measured. The resistin, PAI-I, and adiponectin mRNA expression levels were also measured in the adipose tissue.

**Results:**

Compared to the control group, the FI, FPG, and HOMA-IR scores were significantly lower in the HBOT group, whereas the HDL-C and QUICKI scores were found to be higher. In addition, the resistin, adiponectin, and PAI-I mRNA expression levels were also higher in the HBOT group.

**Conclusion:**

The present study demonstrated that the HBOT had regulated the FI, FPG, and HDL-C associated with metabolic syndrome and diabetes mellitus. Moreover, the study showed that HBOT causes insulin sensitivity by raising adiponectin.

## 1. Introduction

Hyperbaric oxygen therapy (HBOT) refers to a medical application where 100% oxygen is administered for a certain period of time under atmospheric pressure greater than 1.4 [1]. Under normal conditions, approximately 97% of the oxygen in the blood is transported to the tissues in the form of hemoglobin, while 3% is dissolved in the plasma [2]. Since under normal atmospheric pressure, the hemoglobin in arterial blood is nearly saturated with oxygen in healthy individuals, no significant increase occurs in hemoglobin-based oxygen whether breathing 100% oxygen under normal pressure or under HBOT. However, a significant rate of increase in the amount of oxygen dissolved in the plasma (partial oxygen pressure) is observed under HBOT [3]. The partial oxygen pressure in the arterial blood is about 100 mmHg at sea level, whereas it varies according to applied pressure and may increase up to 2000 mmHg under HBOT applications [4].

The HBOT is used to treat medical conditions such as soft tissue infections, radiation damage, thermal burns, sudden hearing loss, and carbon monoxide poisoning [5]. For various reasons, this method of treatment expedites the transition of oxygen to damaged tissues and stimulates collagen synthesis, angiogenesis, the immune response, and stem cell migration. It also accelerates the healing of damaged tissues [6,7].

Many studies have shown the association of high glucose levels with endothelial dysfunction, vascular damage, and endothelium-dependent vasodilation, the association of triglyceride levels with the risk of coronary heart disease (in some studies), and the inverse relationship between high-density lipoprotein cholesterol (HDL-C) levels and cardiovascular disease [8–11]. As for insulin resistance, it may cause metabolic syndrome (MetS), diabetes mellitus (DM), hypertension, and endothelial dysfunction [12].

Adiponectin is abundantly synthesized by adipose tissue. During the differentiation of preadipocytes into adipocytes, its mRNA expression is significantly increased. Low adiponectin levels are associated with insulin resistance, type 2 diabetes, and MetS [13].

The plasminogen activator inhibitor-I (PAI-I) is presented as a circulating peptide of adipose tissue origin because of its strong correlation with body adipose tissue and body mass index. It is an inhibitor of the plasminogen activator, and acts in the development of type 2 diabetes and insulin resistance [14,15].

Resistin is synthesized by many tissues including white adipose tissue. Interestingly, the sites (tissues/cells) of major synthesis in humans and animals are different. Resistin transfusion disrupts glucose tolerance and glucose uptake to adipocytes. Thus, resistin plays an important role in the development of insulin resistance [16].

Wilkinson et al. reported that HBOT caused insulin sensitivity [17], but the reason for this is not yet known. In addition, previous studies have demonstrated that resistin, PAI-I, and adiponectin are associated with insulin resistance/sensitivity [13–16].

According to these findings, we hypothesized that HBOT might cause insulin sensitivity by affecting the expression of resistin, PAI-I, and adiponectin. Therefore, in the present study, we aimed to investigate the effect of HBOT on insulin sensitivity via resistin, PAI-I, and adiponectin. We also aimed to investigate the effects of HBOT on the plasma levels of HDL-C **,** fasting insulin (FI), fasting plasma glucose (FPG), and triglyceride, the abnormal levels of which are risk factors for MetS and DM.

## 2. Material and methods

### 2.1. Animals and treatments

All procedures performed on rats were carried out with the approval of the Local Ethics Committee of Animal Experiments (2017/4/3-2018/4/5). The study was carried out at the Düzce University Experimental Animal Application and Research Center between 3 July 2018 and 20 March 2019. Animals were treated in accordance with the Guide for the Care and Use of Laboratory Animals (8th edition, National Academies Press). The study was performed using two groups of eight male Sprague–Dawley rats weighing 100–160 g. The first group was defined as the control group. The rats in this group were fed and housed under the same conditions as the HBOT group except for the application of HBOT throughout the experiment. The second group was defined as the HBOT group. Unlike the control group, HBOT was applied to this group (2.5 atmospheres, 100% oxygen). Rat feed (Optima, rat 24/27, Turkey) and water were provided ad libitum.

The HBOT cabinet used in this study was specially designed of 2.5-mm steel, holding approximately 19 L and resistant to pressure higher than 2.5 atmospheres (atm). The environment included a pressure-resistant, transparent, plexiglass monitoring window (for checking the health status of the animals), a humidification unit to moisturize the oxygen entering the cabinet, a cabin pressure manometer, and an adjustable automatic valve (for both exhaust and safety).

The HBOT was conducted in the HBOT cabinet at the Düzce University Experimental Animal Application and Research Center. After the rats in the experimental group were placed in the cabinet, initially, the valve of the high-pressure oxygen tube was turned on. The valve of the oxygen regulator was then turned on in order to increase the pressure in the cabinet gradually, so as not to exceed 0.1 atm/min. When the pressure in the cabinet had reached 2.5 atm, a 90-min administration/test period began. For 90 min, 2 L/min of oxygen was passed through the humidifier water in the regulator and into the cabinet. In order to prevent the increasing pressure caused by the oxygen from entering the cabinet, air was discharged through the automatic valve adjusted to 2.5 atm pressure. Thus, the pressure in the cabinet remained constant at 2.5 atm and oxygen circulation in the cabin was ensured. At the end of the 90-min administration/test period, the cabinet pressure was gradually reduced. The reduction was carried out at a rate not exceeding 0.1 atm/min. When the pressure in the cabinet reached normal atmospheric pressure, the animals were removed from the cabinet and placed in their normal feeding area. Finally, the oxygen tube valve was turned off and the process was complete. The same procedure was performed every day for 20 days.

Following 20 days of HBOT administration, the animals were sacrificed by decapitation under anesthesia (Alfamin 90 mg/kg, Alfazin 10 mg/kg) after one night of fasting. After decapitation, blood samples were collected in tubes using a funnel. The abdomen was then immediately opened and retroperitoneal adipose tissue samples were excised. All adipose tissues and blood plasmas were stored at –80 °C until relevant measurements were conducted. The levels of triglyceride, FI, FPG, and HDL-C were measured in these plasma samples.

### 2.2. Measurement of parameters and statistical analysis

The measurements of the rat FPG (66300, Beckman Coulter, USA), HDL-C (ODC0011, Beckman Coulter, USA), and triglyceride (66300, Beckman Coulter, USA) were performed using the autoanalyzer (Beckman Coulter, AU680, USA) at the Düzce University Faculty of Medicine Biochemistry Laboratory. The FI (E-EL-R2466, Elabscience, USA), PAI-I (E0636Ra, Bioassay Technology Laboratory, PRC), and resistin (E-EL-R0614, Elabscience, USA) measurements, on the other hand, were performed at the Düzce University Experimental Animal Application and Research Center using a purchased enzyme-linked immunosorbent assay (ELISA) kit. This was carried out on a microplate reader device (BioTek, 800 TS, USA) according to the kit protocol. 

Total RNA was extracted from the retroperitoneal adipose tissue using NucleoZOL reagent (Macherey-Nagel, Germany). The cDNA was then synthesized from the total RNA using a kit (ProtoScript First Strand cDNA Synthesis Kit, BioLabs, USA). The resistin, PAI-I, and adiponectin mRNA expression levels were measured from the synthesized cDNA with a real-time quantitative polymerase chain reaction (RT-qPCR) kit (Ampliqon, Denmark) on a RT-qPCR device (Roche LightCycler 480 II, Switzerland). The reference gene (Glyceraldehyde 3-phosphate dehydrogenase (GAPDH)), resistin, PAI-I, and adiponectin primer sequences (Oligomer, Turkey) are described as shown in Table. The expression results (measured as fold change) were calculated in accordance with Livak and Schmittgen [18].

**Table:** Gene and primer sequences.

Gene	Primer	Primer sequences
Adiponectin	Left primer	5’TGAAGGGATTACTGCAACCGAA3’
	Right primer	5’CTCCTGTCATTCCAGCATCTCC3’
Resistin	Left primer	5’TGTCCTACATTGCTGGTCAGTC3’
	Right primer	5’TTCTCAATCAACCGTCCTCAGG3’
PAI-I	Left primer	5’CGAAATGTGGTCTTCTCTCCCT3’
	Right primer	5’CGTCCGCAGTACTGATCTCATT3’
GAPDH	Left primer	5’TAGACAAGATGGTGAAGGTCGG3’
	Right primer	5’CTTCCCATTCTCAGCCTTGACT3’

The homeostatic model assessment of insulin resistance (HOMA-IR) and quantitative insulin sensitivity check index (QUICKI) scores were calculated using the data obtained from the FPG and FI measurements according to the following formulae:

HOMA-IR = FI (µU/mL) × FPG (mmol/L)/22.5

QUICKI = 1/[log (FI) +log(FPG)]) [19]. 

Conversion factors: Blood glucose 1 mmol/L = 18 mg/dL and insulin 1U = 0.0347 mg.

The difference between the groups was determined using the nonparametric Mann–Whitney U test. The results of the parameters were expressed as mean ± standard error of the mean (SEM). All analyses were performed at a 95% confidence interval using SPSS 16.0 (SPSS Inc, Chicago, USA), with p ≥ 0.05 accepted as null and P < 0.05 as statistically significant.

## 3. Results

**Figure 1:**
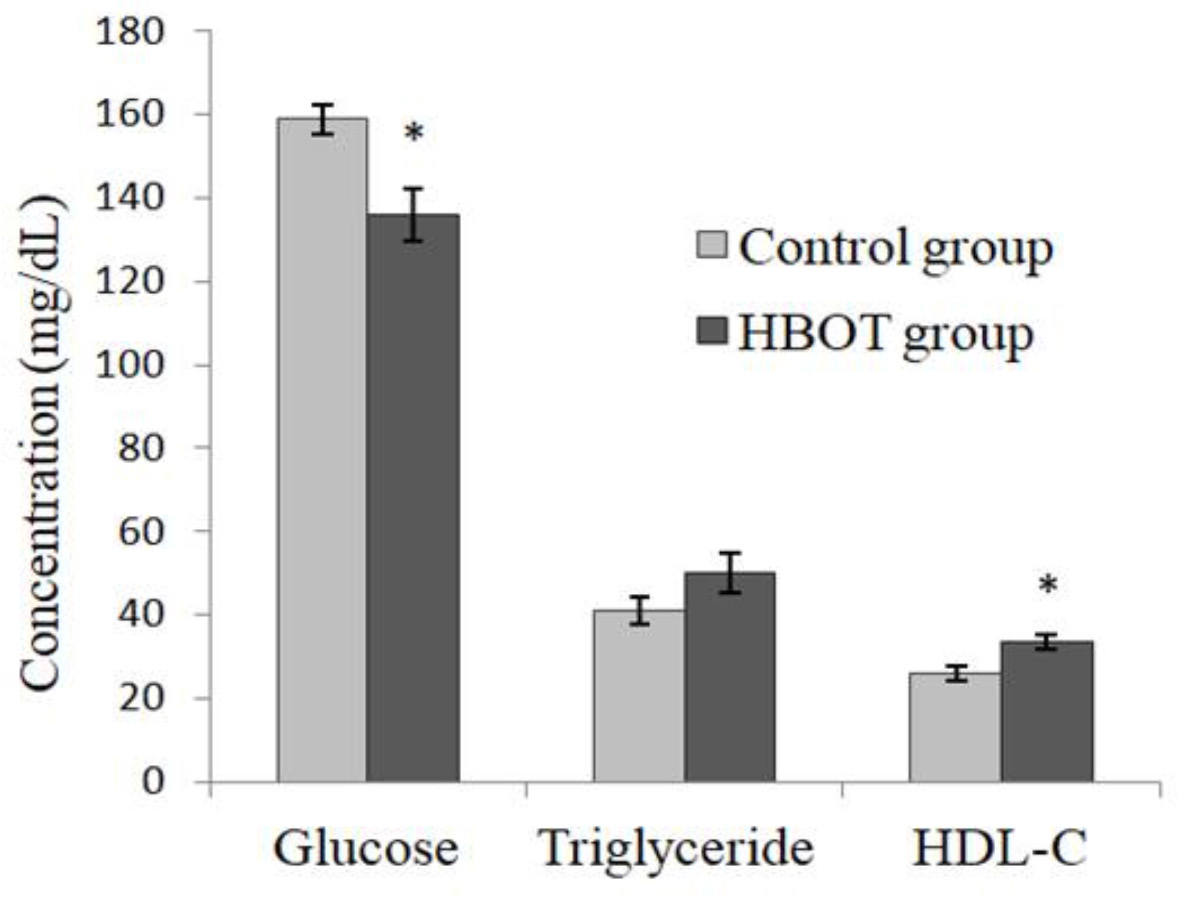
Glucose, triglyceride, and HDL-C levels. *Compared with the control group (p = 0.016, p = 0.141, p = 0.012, respectively). Values are mean   SEM. (HDL-C: highdensity lipoprotein cholesterol; HBOT: hyperbaric oxygen therapy).

After 20 days of HBOT administration, FPG, triglyceride, HDL-C, and FI measurements were performed on the rat blood plasma. The FPG level of the HBOT group was significantly lower than that of the control group (p = 0.016) (Figure 1). When compared in terms of triglyceride levels, no significant difference was seen between the control group and the HBOT group (p = 0.141) (Figure 1).

**Figure 2:**
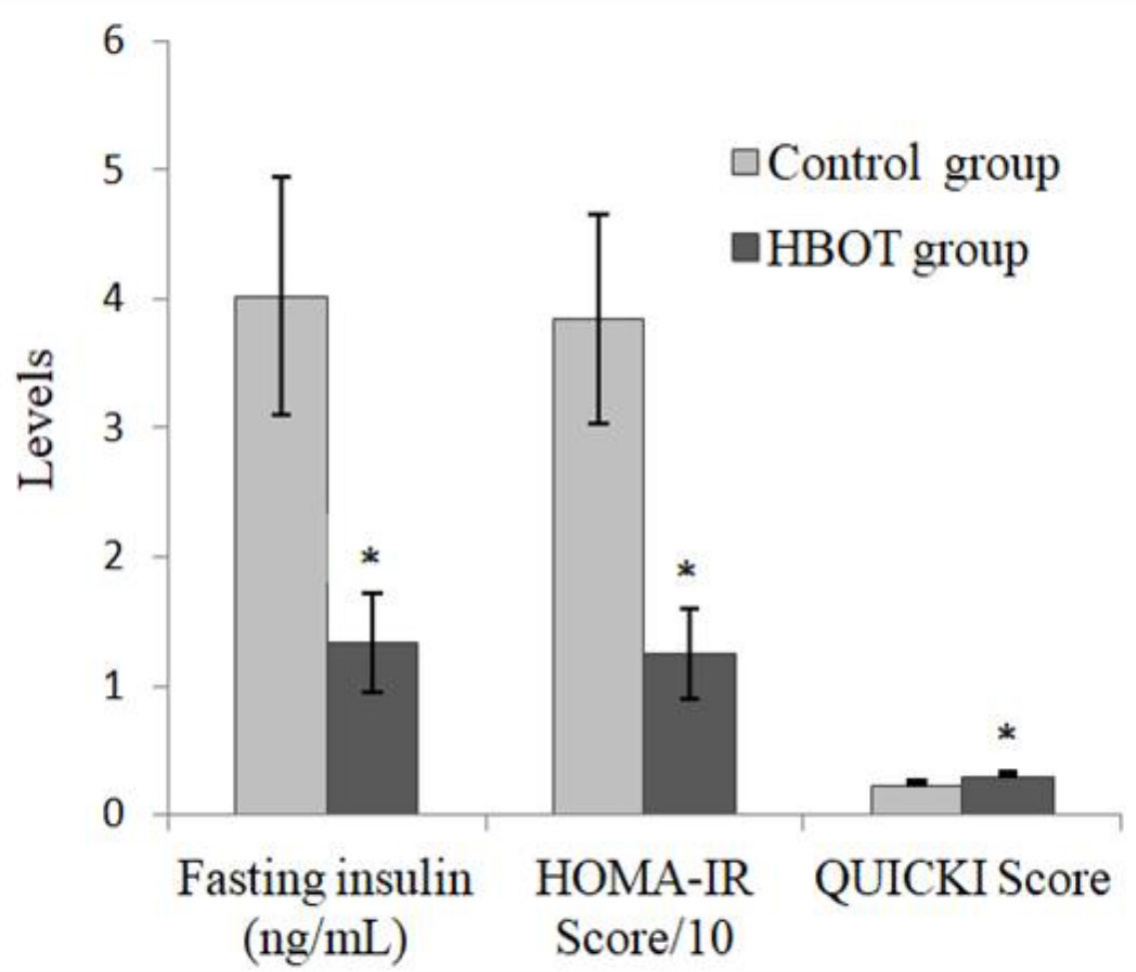
Fasting insulin levels and scores for HOMA-IR and QUICKI. *Compared with the control group (p = 0.013, p = 0.012, p = 0.012, respectively). Values are mean   SEM. (HBOT: hyperbaric oxygen therapy; HOMA-IR: homeostatic model assessment of insulin resistance; QUICKI: quantitative insulin sensitivity check index).

The HDL-C level of the HBOT group was significantly higher than that of the control group (p = 0.012) (Figure 1). The FI levels, on the other hand, were significantly lower in the HBOT group than in the control group (p = 0.013) (Figure 2).

When HOMA-IR was calculated using the FPG and FI level values and compared in the control and HBOT groups, it was discovered to be significantly lower in the HBOT group (p = 0.012). However, the QUICKI scores of the HBOT group were significantly higher than those of the control group (p = 0.012) (Figure 2).

**Figure 3:**
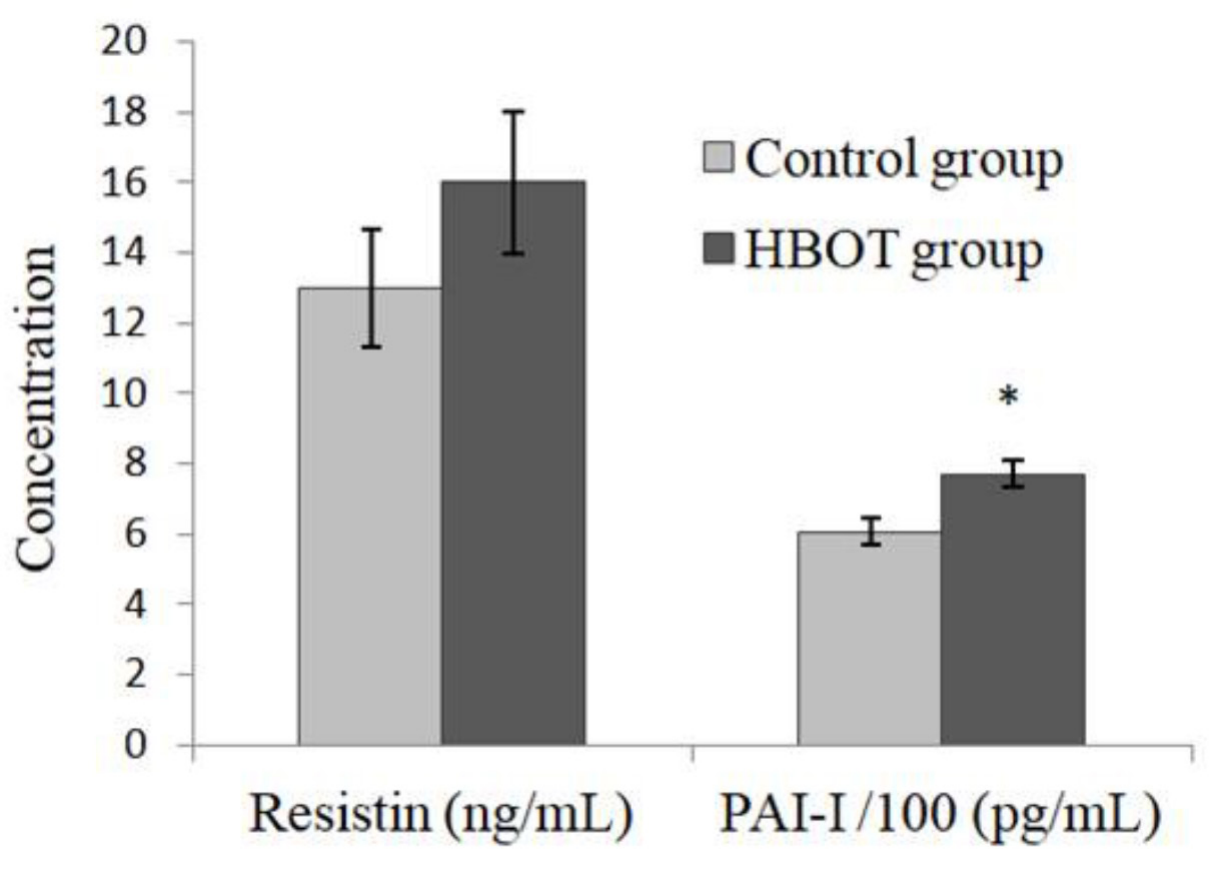
Resistin and PAI-I levels *Compared with the control group (p = 0.257, p = 0.027, respectively). Values are mean   SEM. (HBOT: hyperbaric oxygen therapy; PAI-I: plasminogen activator inhibitor-I).

**Figure 4:**
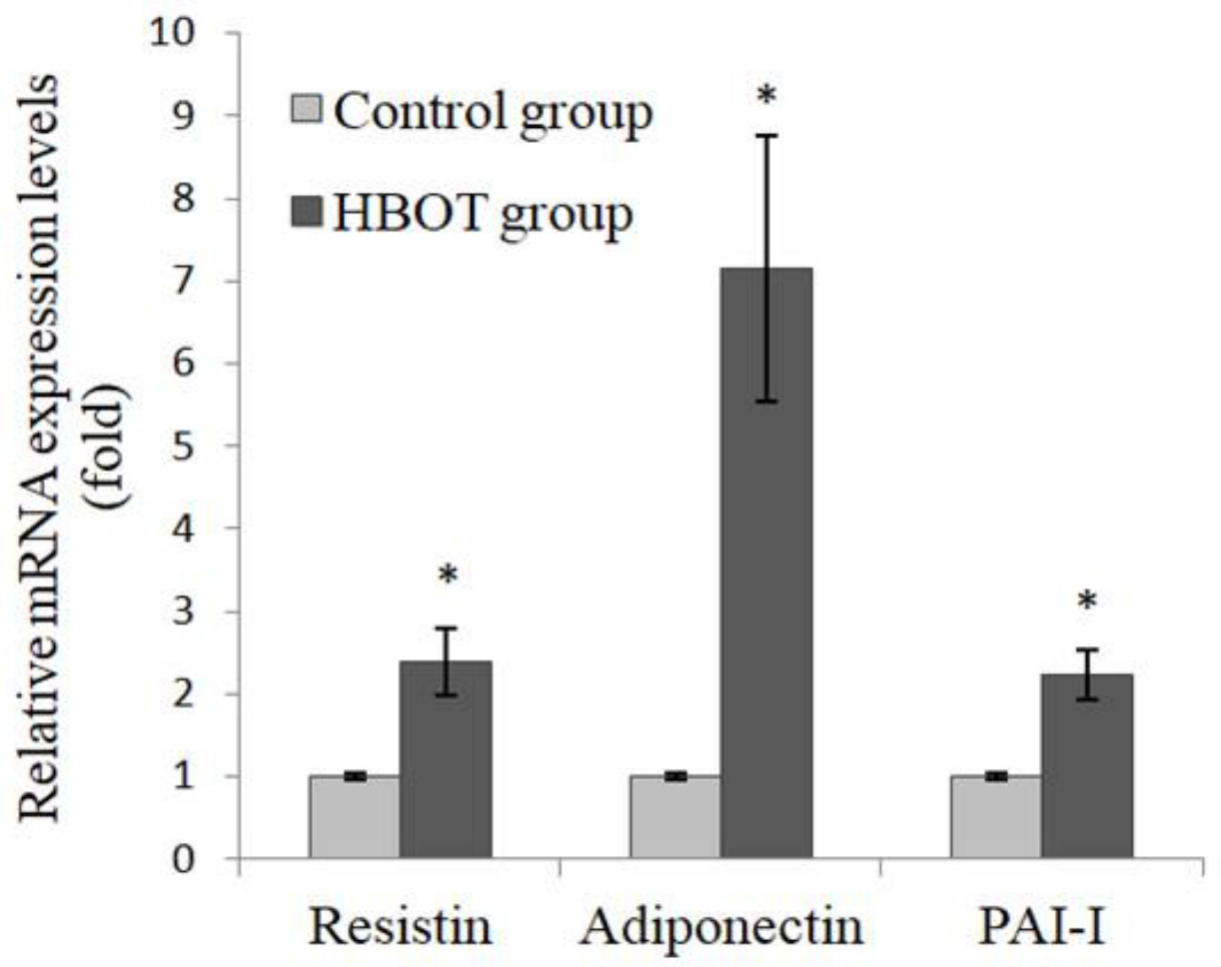
Relative mRNA expression levels in adipose tissue *Compared with the control group (p = 0.002, p = 0.002, p = 0.001, respectively). Values are mean   SEM. (HBOT: hyperbaric oxygen therapy; PAI-I: plasminogen activator inhibitor-I).

The levels of resistin and PAI-I in the plasma of the HBOT group were found to be higher than in the control group (Figure 3). The high level was statistically significant for PAI-I, but not for resistin (p = 0.027 and p = 0.257, respectively). The resistin, adiponectin, and PAI-I mRNA expression levels in the retroperitoneal adipose tissue of the HBOT group were significantly higher than in the control group (p = 0.002, p = 0.002, and p = 0.001, respectively) (Figure 4). 

## 4. Discussion

Glucose is the main energy source of tissues. Blood glucose is obtained via dietary carbohydrates postprandially, via glycogenolysis in short-term fasting, and via gluconeogenesis in long-term fasting [20]. High glucose levels (hyperglycemia) have been associated with cardiovascular diseases [8]. The present study found that the FPG level of the control group was significantly higher than that of the HBOT group (p = 0.016). In previous studies, it was reported that oxygen application (1.25 atm pressure, 36% oxygen) significantly decreased FPG levels in diabetic rats. In a study performed on healthy Wistar rats, the FPG level of the oxygen treatment group was significantly lower than in the control group [21]. Cortisol increases the blood glucose level [22]. Given that HBOT reduces the level of cortisol [23], it could be stated that cortisol reduction might have contributed to the reduced blood glucose levels.

In the present study, the FI level in the HBOT group was discovered to be lower than in the control group (p = 0.013), whereas Yasuda, in his study of healthy Wistar rats, found no significant difference between the HBOT group and the control group in terms of insulin levels [21]. One reason for the different results encountered between that study and ours might be that different animal species were used (Wistar rats vs Sprague–Dawley rats). The metabolic difference between these two species might have caused different results. Secondly, the Wistar rat study was conducted at 1.25 atm pressure and the HBOT was administered for 6 h. In our study, however, the HBOT was administered at 2.5 atm pressure for 90 min. There were also differences in the administration that might have caused the results to differ.

Insulin resistance is defined as the insufficient effect of insulin on tissues [24], and is measured using the HOMA-IR method [19]. Insulin sensitivity is determined by using the QUICKI score [19]. Insulin resistance is an important risk factor in the development of MetS and DM [25]. In the present study, low HOMA-IR scores and high QUICKI scores were detected in the HBOT group (p = 0.012 and p = 0.012, respectively). Because HBOT increases insulin sensitivity, it may prevent the development of MetS and DM.

In the present study, it was found that the HDL-C levels increased significantly in the HBOT group, whereas no change was observed regarding triglyceride levels (p = 0.012 and p = 0.141, respectively). In a study conducted on patients with diabetic foot ulcers, the HDL-C level significantly increased after HBOT administration. On the other hand, there was no significant change in the triglyceride levels of the same patients following HBOT administration [26]. Low HDL-C is frequently seen in MetS and DM [27]. Considering both animal and clinical studies, protective effects against atherosclerosis and coronary heart diseases have been associated with HDL-C levels [28,29]. Because HBOT increases the level of HDL-C, it may reduce the risk of cardiovascular diseases. In addition, it may positively affect the course of MetS and DM.

Both clinical and animal studies suggest that HBOT regulates blood glucose via many signaling pathways or molecules and leads to insulin sensitivity [30–35]. In the mouse study of type 1 DM conducted by Limin et al., it was suggested that with HBOT, more glycogen was stored in the liver, the ratio of the islet beta-cell area to the total islet area increased, the plasma total ghrelin level increased, and the glucose metabolism dysfunction was ameliorated [30]. In an animal study on type 2 DM conducted by Liu et al., it was determined that HBOT increased pancreatic beta cells and both p-Akt and glucose transporter 4 (GLUT-4) expression in the muscle. The GLUT-4 transports glucose into the myocyte, where it is then stored as glycogen and plays an important role in the regulation of glucose homeostasis. Liu et al. reported that insulin sensitivity was induced via HBOT by stimulating the Akt signaling pathway and increasing GLUT-4 expression in the muscle [31]. In the clinical study conducted by Wilkinson et al., HBOT induced acute interleukin-6 (IL-6) increase and insulin sensitivity in the nondiabetic obese group. Since IL-6 infusion increases insulin sensitivity, the increase in IL-6 and insulin sensitivity together was found to be remarkable [32]. In the clinical study conducted by Xu et al., it was stated that HBOT reduced plasma glucose, insulin, and hemoglobin A1c levels and improved insulin sensitivity in diabetic patients [33]. Vera-Cruz et al. determined in their study that HBOT contributed to glucose homeostasis and regulated insulin sensitivity in patients with type 2 DM. The carotid body functions as a sensor of glucose and insulin, and surgical removal of its nerves prevents the development of diet-related metabolic diseases. Therefore, Vera-Cruz et al. reported that HBOT might have caused glucose tolerance by blocking carotid body chemoreceptors [34].

Resistin, PAI-I, and adiponectin are adipokines that are associated with insulin resistance/insulin sensitivity. Resistin and PAI-I cause insulin resistance [35,36]. As for adiponectin, it increases insulin sensitivity [13]. In our study, both resistin and PAI-I mRNA expressions were higher in the HBOT group compared to the control group. Plasma levels of these two parameters versus their mRNAs were statistically high in PAI-I but not in resistin (Figure 3). It is stated that protein expression from mRNA may be repressed in the posttranscriptional process by microRNAs [37]. Although the resistin mRNA expression increased significantly in the HBOT group, its plasma level did not increase significantly. A possible reason for this may be the repression of protein expression from the resistin mRNA by microRNAs. All adipokine mRNA expressions increased in the HBOT group, including the adiponectin and insulin sensitivity that was observed as a result. Although the resistin and PAI-I mRNA expressions were high in the HBOT group, the insulin sensitivity was raised. A possible reason for this may be that the effect of adiponectin is more dominant than the effect of resistin and PAI-I. The increase in adiponectin mRNA was approximately seven-fold in the HBOT group compared to the control group, whereas the increases in resistin and PAI-I mRNA were approximately two-fold (Figure 4). The high rate of increase in adiponectin mRNA supports this possible reason. Adiponectin reduces plasma glucose levels by increasing 5’-adenosine monophosphate-activated protein kinase, peroxisome proliferator-activated receptor gamma, and p38 mitogen-activated protein kinase activities in the liver and muscle. Adiponectin causes insulin sensitivity through these pathways [13]. Therefore, insulin sensitivity may have occurred in the HBOT group.

Our study has limitations. The parameter baseline levels were not measured prior to HBOT. However, the study groups were randomly selected from animals of the same age and size.

The data we obtained in this study demonstrated that HBOT positively affects plasma levels of HDL-C and FPG (reducing FPG levels, increasing HDL-C). In addition, the study demonstrated that HBOT causes insulin sensitivity by increasing adiponectin expression levels and by reducing insulin levels. Thus, HBOT may be administered to prevent the occurrence of insulin resistance and cardiovascular diseases.
